# Medical Applications of Metallic Bismuth Nanoparticles

**DOI:** 10.3390/pharmaceutics13111793

**Published:** 2021-10-26

**Authors:** Catherine Gomez, Gauthier Hallot, Sophie Laurent, Marc Port

**Affiliations:** 1 Laboratoire de Génomique, Bioinformatique et Chimie Moléculaire (EA 7528), Equipe Chimie Moléculaire, Conservatoire National des Arts et Métiers (CNAM), HESAM Université, 2 Rue Conté, 75003 Paris, France; catherine.gomez@lecnam.net (C.G.); gauthier.hallot@nexdot.fr (G.H.); 2 Service de Chimie Générale, Organique et Biomédicale, Laboratoire de RMN et d’Imagerie Moléculaire, Université de Mons, 19 Avenue Maistriau, B-7000 Mons, Belgium; Sophie.LAURENT@umons.ac.be

**Keywords:** bismuth, nanoparticles, theranostic agents, biocompatibility

## Abstract

Recent reviews described the efficient syntheses of metallic bismuth nanoparticles. Nevertheless, few studies have been published on the medical applications of these nanoparticles compared to the number of studies on the well-documented clinical use of the bismuth(III) complex. An analysis of the literature revealed the significant potential of metallic bismuth nanoparticles in different theranostic applications. In the diagnostic field, preclinical proofs of concept have been demonstrated for X-ray, photoacoustic and fluorescence imaging. In the therapeutic field, several preclinical studies have shown the potential of bismuth nanoparticles as X-ray radiosensitizers for use in radiotherapy and as photothermal agents for applications in near infrared phototherapy. The properties of these metallic bismuth nanoparticles as bactericidal, fungicidal, antiparasitic and antibiofilm agents have also been studied. Although information concerning the toxic effects of these nanoparticles has been collected, these data are insufficient when considering the immediate clinical use of these new nanoparticles.

## 1. Introduction

Recently, the synthesis of metallic bismuth nanoparticles (Bi NPs) has been reviewed by our group. Despite the interest in nanoparticle technologies, only approximately fifty papers have described metallic Bi NP production [1], while many works describe the synthesis and biomedical applications of non-metallic bismuth nanoparticles such as bismuth oxyhalides and bismuth chalcogenides, including bismuth oxide, bismuth sulfide, bismuth selenide, and bismuth telluride [2]

Bismuth is a diamagnetic semimetal with a very small band gap. This material shows several interesting properties, such as high magnetoresistance, thermal conductivity and high anisotropic electronic behaviour [3]; these properties prompted researchers to synthesize Bi NPs for electronic applications. Bi NPs have also been studied as chemical catalysts. Recently synthesized Bi NPs have proven to be efficient, when used with NaBH_4_, for reducing 4-nitrophenol [4,5]. On the other hand, Cui et al. characterized the photocatalytic activity of Bi NPs [6,7].

Bismuth(III) complexes are used in medicine. For example, bismuth subsalicylate is used for the relief of diarrhoea and upset stomach due to overindulgence in food and drink. This single-dose medicine contains milligram quantities of bismuth(III) in complex with salicylate. Another bismuth(III) complex, bismuth subcitrate potassium, is used in combination with antibiotics and proton pump inhibitors for the treatment of *Helicobacter pylori* infections.

**Figure 1:**
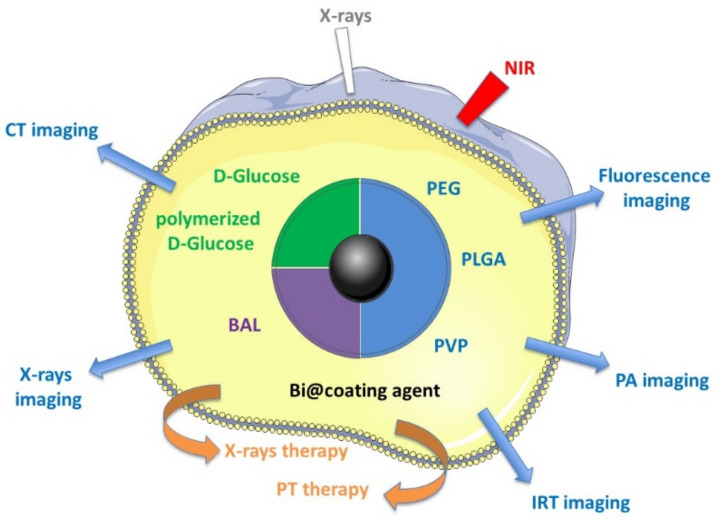
Medical theranostic applications described with different Bi NPs.

Despite this clinical use of bismuth complexes, only a few recently published studies, between 2012 and 2018, have described the medical theranostic applications of metallic Bi NPs, and the purpose of this paper is to provide an exhaustive review of these studies (Figure 1):

## 2. Metallic Bismuth Nanoparticles as Imaging Contrast Agents

The metallic and nanometric properties of Bi NPs have enabled the generation of proofs of concept for their use as contrast agents in different imaging modalities: X-ray, fluorescence and photoacoustic visualization (Table 1).

In general, nanoparticles used for medical imaging are characterized by an increased blood residence time, as their leakage across capillary vessels is limited. Thus, these nanoparticles are well suited for imaging vessels and their abnormalities [8,9,10,11]. 

### 2.1. Metallic Bismuth Nanoparticles as Contrast Agents for X-ray Imaging

Bismuth has the highest atomic number among “nonradioactive elements”, and is characterized by the highest X-ray absorption among the heavy metals at any energy of incident X-ray photons. Consequently, bismuth compounds are attractive for designing new X-ray contrast agents (XCAs). It is particularly interesting that, because of its high atomic number (Z = 83), bismuth has enhanced X-ray opacity compared to that of the clinically approved iodine-based (Z = 53) or barium (Z = 56) XCAs [8]. 

Bismuth oxide (Bi_2_O_3_) and bismuth sulphide (Bi_2_S_3_) nanoparticles have been extensively studied in imaging. However, to be an efficient XCA, a high density of metal atoms must be contained inside the nanoparticle. Therefore, the drawback of Bi_2_O_3_ and Bi_2_S_3_ is the lower concentration of bismuth atoms per particle because of the oxygen or sulphur content. Moreover, the low stability and aggregation tendency of these particles is evident in physiological environments. The instability of Bi_2_S_3_ in aqueous media is problematic because the observed hydrolysis leads to toxic hydrogen sulphide gas. Consequently, metallic Bi NPs are particularly attractive as XCAs because they contain only bismuth atoms, which attenuate X-rays in a relatively small volume and are thus characterized by a high density of atoms opaque to X-rays. Bi NPs are sometimes compared to the well-studied gold nanoparticles (Au NPs) because both are easily synthesized with different sizes and morphologies [12]. However, gold is currently approximately 2000-fold more expensive than bismuth.

The potential of different kinds of radiopaque metallic Bi NPs as high-contrast, long-circulating XCAs was recently explored in four studies.

#### 2.1.1. d-Glucose or Polymerized d-Glucose Coatings

In 2014, the synthesis of Bi NPs coated with d-glucose (Bi@d-glucose) was described [13]. These Bi NPs contain around 6 million bismuth atoms per nanoparticle and are characterized by a very dense bismuth core that constitutes the majority (~60%) of the particle volume. Quantitative computed tomography (CT) using phantoms has demonstrated that Bi@d-glucose NPs have greater X-ray opacity than clinical, iodinated contrast agents (iopamidol, a marketed iodinated XCA) at the same concentration. Moreover, Bi NP attenuation is relatively insensitive to the range of tube voltages used in clinical CT scanners (80 to 140 kV), which is advantageous because the same contrast is produced using any CT imaging protocol. The imaging of cells (HeLa cells and murine macrophages) incubated with different Bi NP concentrations enabled an in vitro quantitative analysis of CT attenuation. CT imaging also revealed that the uptake by both types of cells had a linear correlation with XCA concentration, indicating a nonspecific uptake process. As expected, this uptake was more pronounced with the murine macrophage line, which is consistent with its greater phagocytic activity.

The preparation of highly monodisperse aqueous Bi NPs coated with polymerized d-glucose was reported in 2016 [14]. The CT contrast efficiency of these Bi NPs was evaluated in comparison with that of a BaSO_4_ suspension in vitro. The Bi NPs produced much higher CT contrast per unit of mass concentration than did BaSO_4_, regardless of the CT scanner operating voltage (80 kV and 120 kV). The high stability of these Bi NPs allowed for their oral or rectal administration to mice to achieve CT imaging of the gastrointestinal (GI) tract. After oral administration of these Bi NPs, the upper GI tract and the arrangement of the small intestinal loops were visualized with high contrast. Rectal administration enabled the visualization of the lower GI tract (the rectum and descending colon).

#### 2.1.2. PLGA Coating

Chakravarty et al. studied two kinds of Bi NPs obtained by complex procedures: Bi Ganex (BiG) nanocrystals encapsulated by poly (DL-lactic-co-glycolic acid) (BiG@PLGA) and by a SiO_2_ coating (BiG@SiO_2_) [15]. These two kinds of Bi NPs were dispersed in a 0.5% agarose gel. These phantoms were imaged by a CT scanner operating at a tube voltage of 80 kV and compared with those generated with iopamidol (a marketed iodinated XCA) at various concentrations (0 to 80 mM). The CT contrast enhancement of both Bi NPs was threefold that of Iopamidol (300 mg/mL) at isoconcentration, as demonstrated by the Hounsfield units (HU) quantified with respect to the bismuth or iodine concentration.

Swy et al. used poly (DL-lactic-co-glycolic acid) (PLGA) to encapsulate the Bi NPs [16]. The resulting encapsulated NP had a diameter of 120 nm. After 24 h in an acidic solution imitating the lysosomal medium, the Bi@PLGA NPs showed nearly 70% degradation, whereas in cytosol- and extracellular fluid-imitating media, they remained completely stable. Both a clinical imager and a μCT imager detected these Bi NPs in vitro. The rate of attenuation was higher using μCT because the low-energy component of the μCT X-ray beam was greater than that of the clinical CT system. The ability to detect Bi NPs ex vivo by CT and μCT was also demonstrated by injecting Bi NPs into a chicken wing forearm.

#### 2.1.3. BSA Coating

Bovine serum albumin (BSA)-coated Bi NPs (hydrodynamic diameter of 62 nm) were imaged by CT imaging, operating at a tube voltage of 60 kV [17]. Bi NPs were intravenously injected in mice subcutaneously transplanted with mammary carcinoma tumour cell line 4T1. One hour later, in vivo CT imaging of the mice showed an enhanced contrast of tumour due to Bi NP accumulation, because of the high permeability and retention effect of the tumour. 

### 2.2. Metallic Bismuth Nanoparticles for Dual-Modal Imaging: X-ray and Fluorescence

Bi et al. rendered small Bi NPs water soluble by means of a polyethylene glycol (PEG) coating and used TEM to characterize their diameter as 4 nm [18]. The originality of this work highlights the fluorescent properties of the Bi@PEG NPs and, thus, suggests them as new types of NP for dual-modal X-ray and fluorescence imaging. The spectral emission of the Bi@PEG NPs was studied and a maximum effect was observed at an excitation wavelength of 525 nm. The in vitro CT imaging efficiency was evaluated by determining the HU values. In vivo CT imaging was performed after the intravenous injection of the Bi NPs into mice, with the Bi NPs demonstrating a long circulation time; this property is due to the PEG coating inducing greater Bi NP accumulation in the liver and intestine than was realized with 300 mg/mL iohexol (a marketed iodinated XCA). The in vivo fluorescence imaging was conducted by using a 600 nm excitation wavelength. After Bi NP injection, the fluorescence signal was detected in the chest, epigastrium and, gradually, in the hypogastrium, observations consistent with the CT images.

Bismuth nanoparticles obtained by laser ablation and coated with a non-described surfactant were injected with a micro syringe at a concentration of 1 mg/mL in specific organs of healthy mice [19]. Mice were immediately submitted to X-ray tube irradiation (20–45 kV) in order to provide contrasted fluorescence images acquired with a fast CCD camera. The fluorescence image displays the organ and the nearest blood irrigated tissues very well. This imaging procedure allowed for description of the spatial location of the Bi-NPs as a function of the time from the injection instant.

### 2.3. Metallic Bismuth Nanoparticles for Photoacoustic Imaging

Photoacoustic imaging (PAI) is an interesting non-invasive imaging modality that combines the spectral selectivity of molecular excitation by laser light with the high resolution of ultrasound imaging. The photoacoustic effect is due to the generation of an acoustic wave detected by a transducer and the absorption of optical energy. Compared with fluorescence optical imaging, PAI has a higher spatial resolution (as low as 5 μm) and greater imaging depth (up to 5–6 cm) because the scattering of ultrasonic signals is much weaker than that of light in tissue. Compared with ultrasound imaging, in which contrast is limited by the mechanical properties of the biological tissues, PAI has better tissue contrast, which is related to the optical properties of the different tissues. Metallic NPs, especially Au NPs, have been recently used as PAI contrast agents due to the optical absorption caused by their surface plasmon resonance (SPR) effect. The SPR effect occurs when free charges on the nanoparticle surface oscillate with the electromagnetic field, leading to strong optical absorption.

Four different teams demonstrated that the SPR effect of Bi NPs can generate a signal detectable with PAI [20,21,22,23]. These papers address multimodal therapy and consequently will be analysed in detail in Section 4.2.

All these examples demonstrate that Bi NPs are efficient objects for obtaining multimodal contrast agents. Their intrinsic CT, PA and fluorescence imaging modalities can be combined. However, the proof of concept remains preclinical. The biodistribution and diffusion of these objects is probably limited by their nanoparticulate nature, and will require adjustments to consider their clinical use. 

## 3. Metallic Bismuth Nanoparticles as X-ray Radiosensitizers

Radiotherapy (RT), an effective medical strategy complementary to chemotherapy and surgery that enables the treatment of solid tumours and distant or locoregional metastases, is currently used to treat more than half of cancer patients [24]. The radiation used in RT can indirectly or directly damage tumour cell targets by producing free radicals which induce the increased production of toxic reactive oxygen species (ROS). The difficulties in applying this technique are based on the similar mass energy absorption characteristics of the healthy and cancerous tissues. Improving tumour cell sensitivity to RT remains a major clinical challenge to treating radiation-resistant tumours and to limiting the doses received by healthy organs located near tumours. To sensitize tumours to radiation, NPs consisting of high-Z elements have been demonstrated to function as powerful radiosensitizers in RT during preclinical and clinical trials [24]. Indeed, elements such as gold, platinum, silver, gadolinium, iron and hafnium incorporated into NPs have a large cross-section for radiation absorption and photoelectron or Auger electron generation. These NPs significantly increase the deposited dose in their vicinity because of their high-energy absorption coefficients [12]. Consequently, these metallic NPs are able to concentrate higher radiation doses within tumours, thus enhancing RT efficacy and reducing the risk of possible side effects.

In an initial work, Hossain et al. made a mathematical model to compare the performance of different metallic NPs (bismuth, gold and platinum) as radiosensitizers [25]. Mathematical models quantified the dose enhancement factor, which represents the ratio of the delivered dose to cells with and without NPs. According to this model, Bi NPs provide higher dose enhancements than Au or Pt NPs for a given nanoparticle size, concentration and location. No experimental in vitro or in vivo work was undertaken to support the predictive data in this study.

However, in another paper published in 2012, Hossain et al. described an innovative technique based on the simultaneous use of two kinds of NPs, superparamagnetic Fe oxide NPs and X-ray absorbing Bi NPs. They detected and killed circulating tumour cells released inside the blood stream of patients during cancer development, confronting a major problem in cancer metastasis management [26]. This technique enables the use of an integrated enrichment system, early detection and circulating tumour cell eradication. An in vitro proof of concept demonstrated the feasibility of this approach by simultaneously using the two kinds of NPs, both of which were modified by folic acid ligands that bind to folate receptors overexpressed on tumour cell surfaces. After adding both NPs to the cell suspension, the NPs bound to the surfaces of tumour cells. The use of superparamagnetic Fe oxide NPs allows a micromagnet to be used to localize and immobilize the circulating tumour cells in a small area. Bi NPs enable circulating tumour cells to be detected by X-rays and fluorescence, then radiosensitized, and finally, killed by increasing the X-ray intensity.

Very recently, four teams demonstrated the potential of Bi NPs as radiosensitizers to improve cytotoxic ROS production by radiotherapy [21,27,28,29]. In this section, the work of Deng et al. [27] and Jiao et al. [28] are discussed, while other studies are analysed in Section 4.2.

Metallic Bi NPs coated by cellulose nanofibres (TEM diameter between 2 and 10 nm) were prepared by Jiao et al. [28]. These Bi NPs showed low cytotoxicity when administered alone, but induced concentration-dependent cytotoxicity upon exposure to 10 Gy X-ray radiation, which was indicated by the production of ROS at high yield. To assess the potential of Bi NPs in X-ray radiotherapy in vivo, 4T1 tumour-bearing BALB/C mice were injected intratumorally (100 μL of 2 mg/mL Bi NPs) and then exposed to X-ray radiation (10 Gy). The tumours grew rapidly in the control groups (PBS, PBS with irradiation, Bi NPs without irradiation), but in the presence of Bi NPs under X-ray irradiation, tumour growth was significantly inhibited.

Deng et al. prepared folate-inserted, red blood cell membrane (RBC)-modified Bi NPs (Bi@F-RBC NPs) coated with glucose (TEM diameter: 56 nm) [27]. This particular nanoconstruction allowed for the fine-tuning of the pharmacokinetics, biodistribution and efficacy of the radiosensitizers. Indeed, the incorporation of Bi NPs in RBCs showed a long blood circulation time, whereas folic acid incorporation enabled the targeting of the folate receptor, which is overexpressed in breast cancer. The in vitro X-ray radiotherapy efficiency was demonstrated by the measurement of ROS production (carboxy-H2DCFDA assay) in the 4T1 tumour cells incubated with Bi@F-RBC NPs. A sixfold increase in ROS production was observed in the cells treated with Bi@F-RBC NPs and exposed to X-ray radiation compared to the level observed in the untreated cells. An increase in residual DNA double-strand breaks (H2AX staining) was shown after enhanced X-ray radiation by using Bi@F-RBC NPs. Due to these encouraging results, an in vivo study was conducted on 4T1 tumour cell-bearing BALB/C mice. After intravenous injection of Bi@F-RBC NPs (100 mL of 4 mg/mL Bi NPs) and X-ray irradiation at a dose of 9 Gy, the changes in tumour volume of the mice were monitored. After irradiation, the Bi@F-RBC NPs significantly inhibited tumour growth. The average tumour weight in the mice treated with Bi@F-RBC NPs and exposed to radiation was 6.6-fold lower than that of the mice treated with PBS alone. The in vivo biodistribution and histological analysis indicated that the Bi@F-RBC NPs were excreted from the animal body after 15 days, and no evident damage or inflammation was observed in the major organs.

An original microbiological application of Bi NPs as radiosensitizers was described as significantly damaging to bacterial DNA [30]. Indeed, these Bi NP radiosensitizers were used to induce free radicals and photoelectrons upon X-ray irradiation. The proof of concept of this methodology was demonstrated in vitro on the multidrug-resistant bacterium *Pseudomonas aeruginosa* by using Bi NPs conjugated to a polyclonal antibody specifically targeting bacterial surfaces. After exposure to 40 kVp X-rays for 10 min, no significant harmful effects on human cells (HeLa and MG-63 cells) were observed. Ninety percent of the bacteria were killed in the presence of these Bi NPs (200 mg mL^−1^), whereas only 6% were killed in the absence of Bi NPs.

These different studies clearly show the obvious potential of Bi NPs as radiosensitizers. However, it remains necessary to demonstrate the clinical interest of Bi NPs, in particular in comparison to hafnium oxide nanoparticles (NBTXR3, Hensify ^R^), already authorized for their use in humans to treat soft tissue sarcomas.

## 4. Metallic Bismuth Nanoparticles for Theranostic Applications

### 4.1. Metallic Bismuth Nanoparticles for Dual X-ray Contrast and NIR-Photothermal Therapy (Thermoradiotherapy)

Near infrared (NIR)-based photothermal therapy (PTT) is currently under preclinical and clinical investigation, especially to determine its suitability as a cancer treatment. PTT employs a photothermal agent to generate heat, causing irreversible tumour tissue damage upon NIR laser irradiation. The wavelength of the NIR is minimally absorbed by blood and soft tissues, leading to deep penetration of the laser. Compared to conventional therapeutic modalities, PTT exhibits unique advantages as a cancer therapy, including high specificity, minimal invasiveness and precise spatial-temporal selectivity. The therapeutic efficacy of PTT significantly depends on the transformation of light by photothermal agents to obtain sufficient heat. Metallic NPs composed of gold, silver, palladium or platinum have been described as efficient photothermal agents as, after NIR excitation, they release vibrational energy and, consequently, heat.

Metallic Bi NPs coated with poly (vinylpyrrolidone) (PVP) were used for dual-modal CT and photothermal-imaging-guided PTT [31]. These Bi@PVP NPs could be dispersed in water and showed strong NIR absorbance, a high photothermal conversion efficiency and photostability. The efficiency of these Bi@PVP NPs in CT was demonstrated in vitro, as the Bi NPs produced a higher contrast than iobitridol (a marketed iodinated XCA) at equivalent concentrations. This CT performance was confirmed by CT imaging of UT4 tumour-bearing Balb/c mice intravenously injected with Bi@PVP NPs. Then, in the same animal model, the in vivo PTT effect was demonstrated as a significantly elevated temperature (measured by infrared photothermal imaging at the tumour site). The intravenous injection of 20 mg kg^−1^ Bi@PVP NPs was performed after irradiation with an 808 nm laser (1.3 W cm^−2^). The hyperthermic effect induced by PTT induced complete tumour inhibition at 14 days.

### 4.2. Metallic Bismuth Nanoparticles for Multimodal Imaging and Multimodal Therapy

#### 4.2.1. X-ray Imaging and PTT/RT

The theranostic potential of Bi NPs was demonstrated by evaluating both their diagnostic efficiency in X-ray imaging and their therapeutic efficacy in PTT and RT [29]. For these purposes, uniform and stable lipophilic Bi NPs (TEM diameter: 40 nm) coated with 1-dodecanethiol ligands and encapsulated by amphiphilic PEGylated phospholipid (DSPE-PEG) were prepared and named Bi@SR-PEG NPs. HU quantification demonstrated that these Bi@SR-PEG NPs provided better CT imaging signals than did iobitridol (a marketed iodinated XCA). Subcutaneous 4T1 tumour-bearing mice were intravenously (IV) injected with Bi@SR-PEG NPs (200 mL, 2.0 g L^−1^). CT imaging enabled the detection of the tumour area with a maximum contrast obtained 1 h post-injection, before the washed-out phase. A long-lasting liver contrast enhancement was observed, reflecting the phagocytosis of Bi@SR-PEG NPs by Kupffer cells. Then, Bi@SR-PEG was evaluated first in vitro for its performance in NIR-PTT and RT applications. High NIR absorbance and photothermal efficiency were observed under irradiation with an 808-nm laser (1.0 W cm^−2^) for 10 min. The NIR-PTT potential was confirmed in vitro in the 4T1 cells. After the 4T1 cells were incubated with Bi@SR-PEG NPs (40 mg L^−1^) and exposed to an 808-nm laser (intensity of 1.0 W cm^−2^ for 10 min), a negligible number of stained living cells were observed. The in vitro efficiency of Bi@SR-PEG NPs as X-ray radiosensitizers was demonstrated by using a clonogenic survival assay. After incubation of 4T1 cells with Bi@SR-PEG NPs (40 mg L^−1^) and exposure to X-ray irradiation (4 Gy), the survival fraction of the 4T1 cells was determined to be 0.27 and 0.57 in the presence and absence of Bi-SR-PEG, respectively. The sensitization effect was similar, and these promising in vitro results encouraged the authors to test the synergistic effect of Bi@SR-PEG NPs in NIR PTT and RT in vivo. Subcutaneous 4T1 tumour-bearing mice were intravenously injected with Bi@SR-PEG NPs (200 mL, 2 g L^−1^) and then exposed, successively, to an 808-nm laser with an intensity of 1.0 W cm^−2^ for 10 min and to X-ray irradiation (4 Gy). After 14 days, the mice were sacrificed. This synergistic dual treatment induced the inhibition of tumour growth (determined by measuring the average tumour volume) and cell apoptosis (determined by cell morphology and haematoxylin and eosin staining). Individual therapy (NIR-PTT or RT) had a limited therapeutic effect compared with the synergistic effect of the combined NIR-PTT and RT treatment, as demonstrated in this publication.

#### 4.2.2. X-ray CT/PA Imaging and PTT/RT

Yu et al. described the synthesis and biological evaluation of a multifunctional theranostic agent based on a peptide (LyP-1)-labelled Bi NP (Bi@LyP-1 NP) [21]. The ability to absorb both ionizing radiation and laser radiation ensured that Bi@LyP-1 NPs could be used for dual-modal computed tomography and photoacoustic imaging and the synergistic NIR-photothermal and radiotherapy treatment of tumours. By evaluating the HU values, it was demonstrated that the CT signal enhancement capability of Bi@LyP-1 NPs in vitro was superior to that of iohexol (a marketed iodinated XCA). This was further confirmed in vivo by CT imaging after the intratumoural injection of Bi@LyP-1 NPs into 4T1 tumour-bearing mice. As Bi@LyP-1 NPs enable strong and broad NIR absorption, it was possible to study the potential of the Bi NPs with PAI. After the injection of Bi@LyP-1 NPs intratumorally or intravenously into mice bearing subcutaneous 4T1 cell tumours, high photoacoustic signal intensity was observed in the tumours under a wide range of NIR wavelengths (from 700 to 900 nm). The in vitro performance of the Bi@LyP-1 NPs in NIR PTT and RT was then assessed. The Bi@LyP-1 NPs exhibited a broad absorption, between 200 and 2000 nm, and were capable of acting as NIR PTT agents when irradiated with a near-infrared (NIR-II) laser. The use of an NIR-II laser enabled deeper penetration and higher maximum permissible exposure, which increased the photothermal efficacy, especially in humans in the clinic. The in vitro performance was demonstrated when irradiation of a 200 ppm Bi NP solution with a 1064-nm laser produced a hyperthermic effect, with a temperature increase in approximately 17.2 °C, in comparison to that of pure water, which caused a temperature increase in only approximately 2.8 °C. An in vitro clonogenic survival assay demonstrated the ability of the Bi@LyP-1 NPs to enhance the RT efficacy, even at low concentrations and low doses of radiation. The fraction of the 4T1 cells incubated in a 10 ppm Bi@LyP-1 NP solution that survived was dependent on X-ray irradiation doses. The combined PTT and RT effects were studied in vivo after Bi@LyP-1 NPs were IV injected. The surface temperature of the tumours after laser irradiation of 0.6 W cm^−^^2^ with a 1064-nm laser was monitored by NIR imaging. After Bi@LyP-1 NP injection, the tumour temperature rapidly increased to approximately 45 °C and then stabilized, while after a PBS injection, the temperature was found to increase slowly and only to 39.5 °C. This mild photothermal effect resulted only in a delay in tumour growth. However, after irradiation by X-ray and laser irradiation, tumour growth was completely inhibited, demonstrating strong synergistic effects.

#### 4.2.3. CT/PA Imaging and PTT

Multifunctional Bi NPs were developed as theranostic agents for PA/CT imaging and NIR-PTT [23]. The preparation of 1,2-dilauroyl-sn-glycero-3 phosphocholine (DLPC)-coated spherical Bi NPs (Bi@DLPC NPs) was described, and these Bi NPs were characterized by a TEM-determined size of 47 nm and a hydrodynamic size of 162 nm. The photothermal effect was demonstrated in vitro. The temperature of a Bi@DLPC NP (500 μg mL^−1^) aqueous solution increased by 37 °C after 10 min of irradiation with an NIR laser (880 nm, power density of 1 W cm^2^). This kind of irradiation also induced mitochondrial damage by hyperthermia and MDA-MB-231 cell death in vitro, which was likely due to changes in cell membrane permeability.

PA and CT imaging were performed in vitro and in vivo. In vitro, the PA signal at 880 nm increased linearly with the Bi@DLPC NP concentration. A peak PA signal was observed in the MDA-MB-231 tumour cell-bearing mice 6 h after the intravenous injection of the Bi@DLPC NPs. In vitro, the CT signal intensity also linearly increased with the Bi@DLPC NP concentration.

The CT signal was observed in vivo after intratumoural injection of Bi@DLPC NPs (100 μL, 4 mg mL^−1^) into MDA-MB-231 tumour cell-bearing mice and PTT. Then, an in vivo photothermal effect was demonstrated with MDA-MB-231 tumour cell-bearing mice intravenously injected with Bi@DLPC NPs (1 mg mL^−1^) and irradiated with an NIR laser (880 nm, 1 W cm^2^) for 10 min. The thermic infrared images demonstrated that the surface temperature of the tumour area dramatically increased, from 37.4 to 57.7 °C. Cancer cell growth was abrogated by hyperthermia 14 days after treatment.

Physiologically stable PEG-modified polypyrrole-coated Bi Nanohybrids (Bi@PPy-PEG NHs) were recently prepared by Yang Sisi et al. [22]. The Bi@PPy-PEG NH aqueous dispersion revealed a high photothermal conversion capacity and stability after five repeated irradiation cycles with an 808-nm laser (2.0 W cm^−^^2^) completed within 10 min. The high efficiency was attributed to the contribution of PPy, which is known for its excellent photothermal effect. Consequently, highly effective photothermal ablation of cancer cells (4T1 cells) was achieved in vitro. At a concentration of 100 μg mL^−1^, the Bi@PPy-PEG NHs killed more than 90% of the 4T1 cells in 3 min after NIR 808 nm laser irradiation (2.0 W cm^−^^2^). Then, the PTT effect was determined in vivo with tumour-bearing BALB/c mice subjected to NIR 808-nm laser irradiation (2.0 W cm^−^^2^) twenty-four hours after the Bi@PPy-PEG NHs were IV injected.

Follow-up tumour volume measurements demonstrated that tumour growth was efficiently inhibited after irradiation. Furthermore, high-contrast CT/PA dual-modal imaging was demonstrated both in vitro and in vivo (intratumoural injection of Bi@PPy-PEG NHs in tumour-bearing BALB/c mice), showing great potential to provide accurate diagnostic information for antitumour treatment.

Lu et al. recently prepared Bi NPs coated with PEG-2000-2-distearoyl-sn-glycero-3-phosphoethanolamine (Bi@DSPE-PEG NPs), which were used as CT and PAI contrast agents and PTT agents [32]. The performance of the Bi@DSPE-PEG NPs as CT and PAI contrast agents was demonstrated with phantoms (HU measurement and PA signals as functions of Bi concentration) and in vivo by measurement of PA and CT signals after the Bi NPs were IV injected into the C6 glioma tumour-bearing mice. Photothermal therapeutic performance was demonstrated. The temperature of the Bi@DSPE-PEG NP solution (1 mg mL^−1^) rapidly increased to 70 °C within 4 min under 808-nm laser irradiation at a power density of 1.0 W cm^−2^. The same laser irradiation conditions were used to confirm this photothermal in vivo with C6 glioma tumour-bearing mice with Bi@DSPE-PEG NPs (200 μL of 10 mg mL^−1^ Bi NPs IV injected). Indeed, the tumour volume was significantly reduced 12 days after this PTT treatment.

#### 4.2.4. CT/IRT Imaging and Chemo-Photothermal Therapy (CPTT)

Several Bi NPs have been developed with coatings containing natural biomolecules such as gelatin GEL (BGPs) [33]. These Bi NPs showed CT/infrared thermal (IRT) imaging capabilities as well as antitumor effects under CPTT in vitro and in vivo. After 15 min irradiation with an 808-nm laser (1.3 W cm^−2^), a strong increase in temperature is observed, up to 78.6 °C for a BGP suspension of 500 µg mL^−1^, showing the significant hyperthermia induced by Bi NPs compared to a deionized water solution. After five cycles of on–off irradiation, BGPs demonstrated an excellent photostability. The PTT effect was evaluated in vitro after 5 min irradiation on HepG-2 and HeLa cells with different BGP concentrations. Cell viability decreased below 10% with 500 µg mL^−1^ of BGPs after irradiation while it remained almost unchanged (90%) without irradiation. CT and IRT imaging were then performed on tumour-bearing mice, after IV injection of BGPs (20 mg kg^−1^) and exposure to NIR laser (808 nm, 1.3 W cm^−2^) for 5 min. CT imaging demonstrated that BGPs were excellent contrast agents, while IRT imaging measured a rapid temperature rise to 58 °C, demonstrating a favourable photothermal efficiency of BGPs on HepG-2 tumour cells. In vivo, after 24 h of IV injection of a mixture of BGPs and doxorubicin and under NIR laser irradiation, a significant growth inhibition of HepG-2 tumour cells was observed.

#### 4.2.5. CT/PA/IRT Imaging and PTT

Pegylated metallic Bi NPs (Bi@PEG) were used to produce an in vitro and in vivo proof of concept of their use in trimodal imaging (CT, PA and infrared thermal (IRT)) and antitumour PTT [20].

The performance of the Bi NPs in CT imaging was demonstrated in vitro (HU measurements) and in vivo after they were IV injected into HeLa tumour cell-bearing BALB/c mice. After injection, the signal in the tumour increased gradually and considerably, with a burst 6 h post-injection. A linear PA signal was observed with the increasing aqueous concentration of the Bi@PEG NPs, indicating adequate PA imaging ability of the Bi NPs in vitro. PA imaging was investigated in vivo on tumour-bearing Balb/c mice after Bi NPs were IV injected. The PA signal in the tumour was enhanced and lasted up to 24 h, with the strongest signal observed 6 h after injection. The high imaging contrast and favourable residence time in the tumours make Bi NPs excellent contrast agents for both CT and PA imaging in vivo.

The in vitro PTT effect was confirmed by measuring the number of HeLa cancer cells that died after being incubated with Bi@PEG NPs and irradiated with a laser (808 nm, 1.0 W cm^−2^) for 10 min. The majority of the cells were destroyed under these conditions. The in vivo antitumour PTT was investigated in the HeLa tumour-bearing BALB/c mice after the Bi@PEG NPs were IV injected, and they were subjected to irradiation (808 nm, 1.0 W cm^−2^). During irradiation, real-time IRT imaging was used to monitor the tumour temperature change at different times. With laser irradiation, the tumour temperature rapidly increased and reached a plateau of ~51.6 °C. This temperature was sufficient to inhibit further tumour growth and to ablate the tumour after 10 days.

#### 4.2.6. CT/MRI Imaging and Chemo/Photothermal/Chemodynamic Therapy

Stable Bi nanoparticles (NPs) coated by mesoporous silica (Bi@mSiO_2_@MnO_2_/DOX) were synthetized using a stepwise reaction protocol and a further loading with doxorubicin (DOX) [34]. In vitro, after laser irradiation (808 nm, power density, 1 W cm^−2^), Bi@mSiO_2_@MnO_2_ reached a stable photothermal effect with a high photothermal conversion efficiency of 50%. It was also demonstrated in vitro that MnO_2_ incorporated in the coating of these Bi NPs was able to catalyse H_2_O_2_ decomposition in O_2_. These Bi NPs are also efficient contrast agents for X-ray CT imaging of tumours with a high CT value (6.865 HU mM^−1^). The authors propose a sequential and synergistic mechanism of tumor cell destruction. First, after IV injection, Bi@mSiO_2_@MnO_2_/DOX accumulate in the tumour site. (NIR) light irradiation of Bi NPs induces hyperthermia and a PTT effect. The generated heat triggers the release of DOX and induces a chemotherapeutic effect. The chemotherapeutic effect is then enhanced due to the generation of O_2_ by decomposition of endogeneous H_2_O_2_ by MnO_2_. This production of O_2_ consumes glutathione to produce Mn^2+^, which provide a magnetic resonance imaging signal. Moreover, the presence of H_2_O_2_ and Mn^2+^ in the tumor site also produce toxic hydroxyl radicals, inducing a chemodynamic therapy effect. This clever and rational design of a theranostic Bi NP results in an excellent global performance, as Bi@mSiO_2_@MnO_2_/DOX-injected mice group treated with laser irradiation for 10 min exhibited a complete tumour elimination after 16 days, without any recurrence.

All these examples show the significant potential of Bi NPs to create versatile multifunctional theranostic nanomedicines. It is possible to design nanoparticles that are visible to several complementary imaging technologies in terms of spatial and temporal resolution and contrast. It is also possible to combine several types of synergistic treatment, in particular in oncology. However, the design of these nanoparticles remains complex and will require significant efforts to make their synthesis reliable in order to comply with cGMP drug manufacturing standards.

## 5. Metallic Bismuth Nanoparticles as Bactericidal, Fungicidal, Antiparasitic and Antibiofilm Agents

The increased resistance of microorganisms to common antibiotics has become an important issue in medicine. Indeed, the absence of new alternatives to efficiently treat multidrug-resistant pathogenic bacteria has become a true clinical problem. Some metallic NPs, especially Au and Ag NPs, have been evaluated as new broad-spectrum antimicrobial drugs. Moreover, nanoparticle-based approaches are increasingly sought to develop disinfection systems, self-cleaning surfaces and, especially, to inhibit the growth of biofilms, which is an important concern for both the water treatment and medical sectors. Similar to the research on Au and Ag NPs, few studies have evaluated the ability of either hydrophilic or hydrophobic metallic Bi NPs to inhibit bacterial growth and the formation of biofilms (Table 2).

### 5.1. Bi Citrate NPs

The antimicrobial activity of hydrophilic Bi NPs coated with citrate (Bi@citrate) on *Streptococcus mutans* growth was determined [35]. These Bi@citrate NPs significantly reduced the number of bacteria by 69% (similar to a 0.12% chlorhexidine solution). The minimal inhibitory concentration (MIC) of these Bi@citrate NPs against *Streptococcus mutans* growth was 0.5 mM.

The inhibition of *Streptococcus mutans* biofilm formation was determined by fluorescence microscopy. The results showed a complete inhibition of biofilm formation with a chlorhexidine solution (0.12%, positive inhibition control) and with the Bi@citrate NPs (2 mM in Bi); of the cells, 69% were inactivated, and the number of surviving cells was insufficient to form a biofilm. The authors concluded that these Bi@citrate NPs could be an interesting antimicrobial agent to be incorporated, for example, into an orally administered antiseptic preparation.

### 5.2. Bi Subnitrate NPs

The antibacterial activity of Bi@subnitrate NPs was studied against *Helicobacter* *pylori* strains [36]. These Bi NPs were obtained by biosynthesis through the inoculation of bismuth subnitrate into a culture of pure *Serratia marcescens*. The MIC of the Bi@subnitrate NPs ranged from 60 to 100 μg mL^−1^ for the different *Helicobacter* *pylori* strains. The Bi@subnitrate NP effects were investigated by a ^1^H NMR metabolite footprinting method. The concentrations of the different small metabolites in the *Helicobacter* *pylori* culture medium were increased in the presence of the Bi@subnitrate NPs. These results indicated that Bi NPs interfered with vital biochemical pathways in the strains.

### 5.3. Bi Subsalicylate NPs

The antimicrobial properties of the bismuth subsalicylate (BSS) NPs, synthesized by pulse laser ablation against four opportunistic pathogens, *E. coli*, *P. aeruginosa*, *S. aureus* and *S. epidermidis*, were reported [37]. These pathogens are implicated in chronic wounds, orthopaedic-implant infections, the biofilm growth found on explanted orthopaedic devices and nosocomial infections. The antibacterial effect was determined by using the inhibition ratio of the bacterial growth after 24 h (cell viability was measured by MTT assay). The results showed that the BSS NP size and/or concentration had an impact on the inhibition ratio of *E. coli* and *S. epidermidis*, but not on the more sensitive *P. aeruginosa* or *S. aureus*. Nevertheless, all the BSS NPs efficiently inhibited the bacterial growth of the four pathogen strains in superior ratios to the antibiotic control (ciprofloxacin). The smaller BSS NPs (20 nm) had a better bactericidal effect.

These BSS NPs have also been evaluated as potential antimicrobial agents against several oral anaerobic bacteria from the dental plaque, such as Gram-positive bacteria (*Actinomyces israelii*, *Parvimonas micra*, *Streptococcus mutans*, *Streptococcus sanguinis*) and Gram-negative bacteria (*Aggregatibacter actinomycetemcomitans* serotype b, *Capnocytophaga gingivalis*, *Eikenella corrodens*, *Fusobacterium nucleatum* subsp. *nucleatum*, *Porphyromonas gingivalis*, *Prevotella intermedia*) [38]. The Gram-positive strains showed a maximum inhibition of 60%, while the Gram-negative strains reached up to 90% inhibition. After 24 h of exposure to 1.9–21.7 μg mL^−1^ of BSS NPs, the XTT–MMS assay was performed on each strain. The BSS NPs were the most effective in inhibiting the growth of the following bacteria, *A. actinomycetemcomitans* (90%), *C. gingivalis* (90%) and *P. gingivalis* (91%), at a concentration of 21.7 μg mL^−1^.

### 5.4. Bi PVP NPs

Recently, Bi NPs coated by polyvinylpyrrolidone (PVP) have been tested to evaluate the antimicrobial activity against *Staphylococcus aureus* and *Candida albicans* [39]. After 24 h of incubation at 37 °C, the MIC was measured at 1 μg mL^−1^ for *Staphylococcus aureus*, similar to antimicrobial drugs such as Vancomycin (MIC = 1.5 μg mL^−1^) and Ceftaroline (MIC = 0.5 μg mL^−1^), but better than silver NPs (MIC = 4.0 μg mL^−1^) and copper NPs (MIC > 28.6 μg mL^−1^). Against *Candida albicans*, Bi NPs, with a MIC of 16.0 μg mL^−1^, they were less potent than antifungal agents such as amphotericin B or echinocandins and silver NPs (MIC = 2.0 μg mL^−1^), but they were better than copper nano antibiotics (MIC > 150 μg mL^−1^). The Bi@PVP NPs were also described as the most potent agent in inhibiting the biofilm formation of *Staphylococcus aureus*, with an IC_50_ equal to 1.06 μg mL^−1^, but less potent in the case of *Candida albicans*, with an IC_50_ of 7.9 μg mL^−1^. Scanning electron microscopy confirmed that Bi@PVP NPs hinder the ability to form biofilms and altered cell morphology in both *Staphylococcus aureus* and *Candida albicans*.

### 5.5. BisBal NPs

The bactericidal, fungicidal, antiparasitic and anti-biofilm properties of Bi NPs coated with lipophilic dimercaptopropanol NPs (BisBAL NPs) have been extensively studied in several papers.

The antibacterial activity of BisBAL NPs was determined on *Streptococcus mutans* and *Streptococcus gordonii* strains. The BisBAL NPs inhibited the growth of both strains by more than 70% at 0.1 μM, owing to a twelve-thousand-fold higher effectiveness compared to that of chlorhexidine (1.2 mM) [40]. The MIC of the BisBAL NPs for *Streptococcus mutans* and *Streptococcus gordonii* was 5 μM. The antimycotic activity of BisBAL NPs was studied on *Candida albicans*; the MIC against this fungus was determined to be 10 μM. In a mixed culture of *Streptococcus mutans*, *Lactobacillus* *casei*, *Streptococcus gordonii* and *Candida albicans*, a near-complete inhibition of biofilm formation was observed by using a 100 μM BisBAL NP solution added at 0 h or 18 h post-inoculation. The antimicrobial mechanism of BisBAL NPs has not been completely determined. It is hypothesized that BisBAL NPs interact with the plasmatic membrane of the microbes, altering their permeability and then acting on key enzymes, altering their metabolism and finally causing cell lysis.

In another study [41], the effect of BisBAL NPs on the growth, attachment and biofilm formation of *Pseudomonas aeruginosa* was investigated. This biofilm-forming bacterium is common to both water treatment systems and medical devices. The MIC value was evaluated at 12.5 μM for the complete BisBAL inhibition of bacterial attachment to the track-etched polycarbonate membrane surfaces and lysis of the bacteria embedded in biofilms within 1 h of exposure. The mechanism of antibacterial and antibiofilm activity was not demonstrated. It was proposed that the efficiency of the BisBAL NPs is linked to their lipophilic nature, which favours the association of the NPs with cell wall-bound lipids of the bacteria. A subsequent release of bismuth ions, which combine with the sulfhydryl groups of key respiratory chain enzymes, inactivates and lyses the bacteria. These results showed that the BisBAL NPs are promising antibacterial and antibiofilm agents for water purification applications as well as for the synthesis of antimicrobial surfaces.

The antiparasitic activity of BisBAL NPs on *Trichomonas vaginalis* was recently studied by Rodríguez-Luis et al. [42] *Trichomonas vaginalis* is the major cause of vaginitis, cervicitis and urethritis in women worldwide. New treatments are expected, because 5% of the trichomoniasis clinical cases are caused by parasites resistant to the reference drug (metronidazole). Cell viability assays demonstrated that, after 24 h of BisBAL NP (500 g mL^−1^) exposure, the parasitic growth of *Trichomonas vaginalis* was inhibited by 94.3%. This result was confirmed when no living flagellates were observed by fluorescence microscopy under the same experimental conditions.

Until now, only chlorhexidine, a powerful antiseptic against microbial plaque, is able to eliminate *Enterococcus faecalis*, which is responsible for persistent apical lesions after root canal treatment, but its long-term use increases the risk of oropharyngeal cancer [43]. This bacterium, the most important species in dental diseases, has shown a high persistence and resistance. An alternative treatment with Bi NPs was thus proposed to eradicate it. The BisBAL NPs showed eight times the activity of chlorhexidine. After 24 h of incubation at 37 °C, the MIC was recorded at 5 μg mL^−1^ and the MBC at 10 μg mL^−1^ for BisBAL NP against 40 μg mL^−1^ and 80 μg mL^−1^, respectively, for chlorhexidine.

A similar strategy was used to eliminate *Streptococcus salivarius* [44]. The BisBAL NPs were even better at reducing the biofilm formed by this bacterium. Indeed, the MIC was measured at 2.5 μg mL^−1^ and the MBC at 5 μg mL^−1^ compared to chlorhexidine 12% (MIC: 50 μg mL^−1^, MBC: 50 μg mL^−1^).

It was concluded that BisBAL NPs showed a higher antibacterial activity and lower side effects than chlorhexidine, suggesting an interesting alternative to combat these bacteria, *Enterococcus faecalis* and *Streptococcus salivarius*.

The use of BisBAL NPs in dentistry applications was investigated by studying the antimicrobial, antibiofilm and mechanical properties of mineral trioxide aggregate (MTA) supplemented with BisBAL NPs [45]. The antibacterial and antifungal activities were determined by disc diffusion assay, while antibiofilm activity was analysed by fluorescence microscopy. The MTA supplemented with BisBAL NPs inhibited the growth of *Enterococcus faecalis*, *Escherichia coli* and *Candida albicans,* which showed that BisBAL NPs conferred effective bactericidal and antimycotic properties to the MTA. BisBAL NPs also conferred efficient antibiofilm activity to the MTA and detached the biofilm of fluorescent *Enterococcus faecalis* completely 24 h after treatment, an outcome not accomplished by MTA alone. The microhardness and surface roughness of the MTA supplemented with BisBAL NPs were not significantly different from these features in the non-supplemented MTA. It was concluded that MTA supplemented with BisBAL NPs could fight potential reinfections after endodontic treatment, as this new material composite exhibited antimicrobial and antibiofilm activities without affecting the mechanical properties of the native MTA [45].

### 5.6. Miscellaneous

The antimicrobial activity against several microorganisms was studied with synthesized Bi NPs of average size 40 nm with an undetermined coating [46]. Under in vitro conditions, a high antimicrobial activity was detected against the tested pathogens such as *Campylobacter jejuni* Pl—09.c, *Listeria monocytogenes* ATCC 19112, *Yersinia enterocolitica* 12/15-08, *Salmonella typhimurium* N°16, *Escherichia coli* N°4, *Mycoplasma arginini* G 230, *Acholeplasma laidlawii* ATCC 23206 and *Bacillus anthracis* M-71 microorganisms. Indeed, a total inhibition of the growth of all microorganisms was observed for a Bi NP concentration of 6.5 mg mL^−1^ and 12.9 mg mL^−1^ by metal. Each pathogen was introduced with a seed-dose of 10^3^ to 10^6^ CFU.cm^3^, except for *Mycoplasma arginini* G 230 (10^5^ CFU.cm^3^), *Acholeplasma laidlawii* ATCC 23206 (10^5^ CFU.cm^3^) and *Bacillus anthracis* M-71 (10^7^ CFU.cm^3^). The microorganism *Leptospira Pomona*, on the other hand, required a Bi NP concentration range between 4.8 mg mL^−1^ and 19.4 mg mL^−1^ by metal to inhibit the pathogen growth.

These Bi NPs were characterized as noncytotoxic, nongenotoxic, nonmutagenic and biosafe according to the “Safety assessment of medical nanopreparations” guidelines.

The discovery of new anti-microbial agents is an essential public health issue, not only for antimicrobial treatment, but also as a prevention measure to avoid infection due to treatment. The studies carried out with different Bi NPs are promising but further biological studies are required to demonstrate their potential before considering clinical applications. It will also be necessary to compare the performance of Bi NPs with other types of nanoparticles (in particular silver nanoparticles).

## 6. Biocompatibility and Toxicity of Metallic Bismuth Nanoparticles

The in vitro and in vivo toxicity of several Bi NPs has been evaluated in various papers (Table 3).

Some authors [13,27] have proposed that Bi NPs can be biodegraded by oxidation and dissolution under physiological conditions (i.e., pH < 6, compatible with lysosome pH) and then discharged from the body as soluble bismuth(III) ions, allowing efficient clearance of bismuth ion species. Bismuth(III) ions are considered biocompatible because they are used in medicine and administered at high doses. 

Consequently, the instability of Bi NPs and the metabolism-induced conversion into soluble bismuth(III) species could be a main advantage in terms of long-range toxicity.

### 6.1. Coating of d-Glucose and Its Derivatives

The in vitro cytotoxicity of Bi NPs coated with cellulose nanofibres was evaluated by MTT assay on mouse breast cancer cell lines (4T1 cells). In this assay, the Bi NPs showed excellent biocompatibility at concentrations as high as 150 μg mL^−1^ [28].

The biocompatibility of the Bi NPs coated with d-glucose and 1,2-propanediol was studied through cell viability experiments using MTS assays [13]. After one hour of incubation with Bi NPs, no decrease in viability was observed for HeLa cells or J774.A macrophages. After 24 h of incubation with Bi NPs, no viability decrease was observed for the HeLa cells at concentrations between 0.006 and 0.5 mg Bi/mL. However, macrophage viability decreased with increasing Bi NP concentration, with an apparent LD50 of 50 μg mL^−1^ (0.2 mM).

Wei et al. [14] reported the cytotoxicity and in vivo toxicity of Bi NPs coated with polymerized d-glucose. These Bi NPs were stable after three days of incubation at pH 2.2, simulating the body fluids in the stomach, and pH 8.0, simulating those in the small intestine. According to the results from an MTT assay, the Bi NPs did not influence the viability of the HeLa cells exposed at a concentration of 200 mg L^−1^. The in vivo toxicity was assessed after 5.6 mg of Bi NPs were orally administrated to mice. The mice were sacrificed 7 or 14 days after Bi NP administration, and haematoxylin and eosin were used to stain the mouse digestive organs (stomach, small intestine and large intestine), which showed no significant differences compared with those of the PBS control group and the other Bi NP groups. The liver and kidney function biomarkers were all normal, indicating low levels of toxicity induced by the Bi NPs in these organs.

### 6.2. Polymer Coatings

#### 6.2.1. PEG

The toxicity induced by the Bi@SR-PEG NPs in blood cells was also evaluated by an in vitro haemolysis assay. At concentrations as high as 1000 mg L^−1^, no haemolysis was induced by these Bi@SR-PEG NPs. The in vivo toxicity induced in healthy Balb/c mice by IV injected Bi@SR-PEG NPs (200 mL, 2.0 g L^−1^) was assessed. On days 7 and 28 post-injection, no modification of liver function markers (aspartate aminotransferase and alanine aminotransferase), kidney function markers (urea nitrogen and albumin/globin ratio) or blood formulation was observed in comparison to these indicators in the control group [29], showing the blood compatibility of these NPs.

The cytotoxicity induced by the Bi@PEG NPs [18] was assessed in vitro. L929 cell viability reached 92% when these cells were incubated with Bi@PEG NPs (7.813–500 μg mL^−1^) for 24 and 48 h. In the same study, at a concentration up to 500 mg mL^−1^, no obvious haemolysis due to these Bi NPs was detected.

The Bi NPs coated with different coating agents, PVP, amine, PEG and silica, were compared in terms of cytotoxicity on two cell lines, HeLa and MG-63 cells, with three assays (MTT, G6PD and calcein AM/EthD-1) [47]. Whatever the Bi NPs tested, no cytotoxicity was observed at a concentration of 0.5 nM. At a higher concentration (50 nM), Bi@SiO_2_-NH_2_, Bi@PVP, Bi@SiO_2_ and Bi@SiO_2_-PEG NPs killed 52, 45, 41 and 34% of HeLa cells, respectively, and 33, 29, 24 and 22% of MG-63 cells, respectively. These assays highlighted that HeLa cells were more vulnerable to the cytotoxicity of modified Bi NPs compared to MG-63 cells. However, these Bi NPs were less toxic than the CdSe/ZnS NPs tested, and comparable to iron oxide nanoparticles. This study then showed how all Bi NPs damaged the cells during the cytotoxicity tests. The use of three toxicity assays provides complementary information on Bi NPs cytotoxicity by evaluating the integrity of the cell membrane and activity of the enzyme in mitochondria. Bi@SiO_2_ NPs modify the activity of intracellular esterase but have less effect on mitochondria, indicating that the toxicity of Bi@SiO_2_ NPs is less related to metabolic function. Bi@SiO_2_-NH_2_ NPs affect mainly the metabolic functions of cells, and Bi@SiO_2_-PEG NPs had less effect on cell mitochondria.

The molecular mechanisms (release of metal ions, production of reactive oxygen species) inducing Bi NPs cell damages will require further research to elucidate them.

The cytotoxicity induced by the Bi@PPy-PEG NHs [22] was studied in both normal cells (HUVECs) and cancer cells (4T1 cells) using CCK-8 assays. For both types of cells, the viability was above 90% after 24 or 48 h of incubation with these Bi NPs. After incubation with the Bi@PPy-PEG NHs at a high concentration of 1000 μg mL^−1^ for 24 h, no haemolysis of the erythrocytes was detected.

The Balb/c mice used for a PTT experiment were carefully monitored during the experimental period (eating, drinking, grooming, activity and excretion), and they showed no abnormal signs. Sixteen days after PTT treatment, the major organs were collected and stained with haematoxylin and eosin for the histology analysis. No noticeable inflammation or organic lesions were observed. Furthermore, a serum biochemistry assay was carried out. Liver (Aspartate Aminotransferase (AST), Alkaline Phosphatase (ALP) and Alkaline Phosphatase (ALT)) and renal (uric acid (UA), creatinine (CREA) and blood urea nitrogen (BUN)) function biomarkers were all within normal ranges, suggesting adequate biocompatibility of the Bi@PPy-PEG NHs.

A cell counting kit-8 (CCK-8) assay was used to evaluate the potential cytotoxicity of Bi@PEG-NPs induced in both HeLa cells and human umbilical vein endothelial cells (HUVECs) [19]. After these cells were incubated with Bi@PEG-NPs at concentrations as high as 300 mg mL^−1^ for 24 or 48 h, no observable cytotoxicity was detected in either cell type. Red blood cells (RBCs) in contact with these Bi NPs showed negligible haemolysis. After the Bi NPs were IV injected, no obvious signs of toxic effects, such as changes in eating, drinking, grooming, activity, exploratory behaviour, urination or neurological status, were observed. After mouse sacrifice, no evident organ damage or inflammatory lesions in the major organs were observed by histological examination, and all serum biochemistry parameters, including liver function (AST, ALP, and ALT) and renal function (UA, CREA, and BUN) markers, were within normal ranges.

Bi@DSPE-PEG NPs exhibited no cytotoxicity in C6 and Cos-7 cells [32]. Liver function was measured and blood was analysed after the DSPE-PEG-Bi NPs (200 μL of a 10 mg mL^−1^ Bi@DSPE-PEG NP solution) were IV injected into C6 glioma tumour-bearing mice. The alanine aminotransferase (ALT) and aspartate aminotransferase (AST) levels returned to normal quickly after an initial transient increase. Blood count measurements were within the reference ranges at each time point, and evidence from the histological assay showed no obvious damage in the main organs (heart, liver, spleen, lung or kidney) after the mice received the Bi@DSPE-PEG NP treatment.

#### 6.2.2. PLGA

The biocompatibility of BiG@PLGA (Bi Ganex@PLGA) and BiG@SiO_2_ was studied on the basis of an MTT cell proliferation assay of Raw264.7 macrophages following 24, 48 and 72 h incubation at different Bi NP concentrations [15]. No cytotoxicity was observed in treatments at concentrations as high as 0.01 mg mL^−1^. Then, bismuth(III) dissolution of the BiG@PLGA and BiG@SiO_2_ was evaluated in PBS (pH 7.4) and sodium citrate (pH 5.5), which imitate extracellular and lysosomal fluid, respectively. Over four weeks, bismuth(III) dissolution in the BiG@PLGA and BiG@SiO_2_ NPs was limited to 30% in the sodium citrate and to 3% in PBS solutions. These results indicate a pH-dependent physicochemical degradation that could presage biodegradability after cellular internalization.

The bismuth(III) dissolution rate of the Bi@PLGA NPs was evaluated in PBS (pH 7.4) and in sodium citrate (pH 5.5) [16]. After 48 h, the Bi@PLGA NPs were 100% stable in PBS (mimicking cytosolic and extracellular fluids), whereas the Bi@PLGA NPs showed nearly 70% degradation in the sodium citrate used to mimic lysosomal pH.

MTT cell proliferation assays were performed on STO mouse fibroblasts. No effect on proliferation was detected for Bi NP concentrations that ranged between 0.0001 mg mL^−1^ and 0.01 mg mL^−1^. At the highest concentrations (0.1 mg mL^−1^ and 1 mg mL^−1^), a significant decrease in cell proliferation compared to that of the controls was observed. Flow cytometry was also used to demonstrate that high Bi@PLGA NP concentrations reduced the viability of these cells.

In vivo toxicity studies in rats revealed no clinically apparent side effects after 20 mg kg^−1^ Bi@PLGA NPs was IV injected (24 h) or IP injected (7 days). However, some serum chemistry and haematologic values were significantly altered 24 h and 7 days after NP injection. A pathological and histopathological evaluation was performed, showing transient kidney injury and periportal inflammatory processes in the liver, likely due to the routes of bismuth excretion from the body.

#### 6.2.3. PVP

Recently, Hamood et al. [48] evaluated the anticancer activity of Bi@PVP NPs on the MCF-7 breast cancer cell line. Using MTT cytotoxicity assays, the cytotoxic and antiproliferative effects of the Bi@PVP NPs (7 nM) were demonstrated after 48 h of exposure. Unfortunately, no further work has confirmed this anticancer activity in vivo.

The Bi@PVP NPs described by Lei et al. [31] induced no apparent cytotoxicity in U14 cells after 24 h of incubation, as the cell viability was greater than 90%, even at a high concentration of 500 μg mL^−1^. The blood biochemistry was not modified, and no obvious tissue damage or inflammatory lesion in any major organs (heart, liver, spleen, lung and kidney) was detected two months after these Bi NPs were IV injected at a dose of 20 mg kg^−1^.

### 6.3. BisBAL Coating

The in vitro toxicity induced by BisBAL NPs (lipophilic Bi NPs coated with 2,3-dimercapto-1-propanol) was studied on different cells in different papers.

The cytotoxic effects of the BisBAL NPs were specifically studied on erythrocytes [49], epithelial cells [50], fibroblasts [45] and cancer cells (cervical, prostate, colon in humans).

In the erythrocytes, induced cytotoxicity was studied by performing several in vitro dose response tests for Bi NP concentrations that ranged from 1 to 1000 µM [49].

Based on the results from the cell viability (MTT) assays and the study of erythrocyte morphology using optical microscopy, the cytotoxic effect on erythrocytes was observed for BisBAL NP doses greater than 500 µM. After 24 h of treatment, no damage to the plasmatic membrane of erythrocytes exposed to doses less than 100 µM BisBAL was indicated by an AM calcein assay or fluorescence microscopy. Nevertheless, cell membrane damage was observed at doses greater than 500 µM. Genotoxic assays (comet assays) revealed no damage to the genomic DNA of the blood cells after 24 h of exposure to BisBAL NPs from 1 to 1000 µM. At doses greater than 500 µM and after 24 h of incubation, the BisBAL NPs promoted apoptosis of the blood cells (Annexin V assay). From all these tests, it was concluded that BisBAL NPs at concentrations lower than 100 µM were not toxic to blood cells. However, BisBAL NPs caused damage to erythrocyte plasmatic membranes at a concentration of 500 µM, leading to apoptosis or necrosis in the long term.

The same team studied BisBAL NP-induced cytotoxicity in epithelial cells [50]. No cytotoxicity (MTT test and evaluation of membrane integrity by fluorescence microscopy) was observed in MA104 monkey kidney or HeLa cells cultured with 5 µM BisBAL NPs for 24 h. After 24 h of exposure to 5 µM BisBAL NPs, no DNA damage was detected by genotoxicity assays, comet assay, fluorescence microscopy or electrophoresis. An SDS PAGE assay showed no modification of protein synthesis in the MA104 cells exposed to 5 µM BisBAL NPs. It was concluded that BisBAL NPs do not induce toxic effects in epithelial cells.

The cytotoxicity of MTA supplemented with BisBAL NPs was determined on human gingival fibroblasts (HGFs) [45]. A confluent monolayer of HGFs was exposed to BisBAL NPs, MTA and MTA supplemented with BisBAL NPs for 24 h. After treatment, cells were stained with crystal violet and observed using optical microscopy. Interestingly, the viability of the HGFs treated with MTA supplemented with BisBAL NP was significantly increased compared to that of the other treatment groups.

BisBAL NP hydrogels have recently been studied for cytotoxicity induction in HeLa, DU145 and HCT-116 cancer cells [51]. The inhibition of tumour cell growth was dependent on the duration of exposure to the BisBAL NP hydrogel. After 18 h, HeLa cell growth was inhibited by 99%, DU145 cell growth was inhibited by 82% and HCT-116 cell growth was inhibited by 60% with a 50 μM BisBAL NP hydrogel. As the activity of the BisBAL NP hydrogel was time- and dose-dependent, an increase in the BisBAL NP concentration to 250 μM after 1 h increased the growth inhibition (93% for the HeLa cells, 99% for the DU145 cells and 96% for the HCT-116 cells). The in vitro evaluation showed that the efficacy of the BisBAL NP hydrogel was similar to that of docetaxel and cisplatin on three cancer cell lines, but that the hydrogel induced lower toxicity in noncancer cells. The hydrogel enabled the use of a higher concentration of BisBAL NPs without inducing adverse effects. No cytotoxicity or tissue damage was observed in the organs evaluated in male BALB/c mice 30 days after BisBAL NP treatment (kidney, liver, brain, cerebellum, heart and jejunum). BisBAL NP hydrogels are considered innovative treatments for cervical, prostate and colon human cancer with no side effects on non-tumour cells.

The effect of BisBAL NPs was also evaluated on human MCF-7 breast cancer cells [52]. The proliferation of the MCF-7 cells treated with a high dose of BisBAL NPs (25 μM) was determined to be 81% that of the MCF-10 fibrocystic mammary epitheliocytes used as control cells (24%). Cell membrane permeability (MTT assay) was altered after exposure to BisBAL NPs (100 μM), and 95% of the tumour cells died. At doses above 10 μM, the genotoxicity of the BisBAL NPs was found to promote strand breaks in DNA (comet assay and fluorescence microscopy). At low concentrations, BisBAL NPs induced apoptosis of the tumour cells. The action mechanism of the BisBAL NPs, which was dose-dependent, showed altered tumour cell membranes and damage to genomic DNA.

### 6.4. Other Coatings

Bi@DLPC NPs synthetized by Yang et al. [23] showed negligible cytotoxicity in MDA-MB-231 and MCF-10A cells (breast epithelial cells) (MTT assay) at concentrations below 250 μg/mL after 24 h of incubation.

Bi@LyP-1 NP-induced cytotoxicity was evaluated by using a standard cell counting kit-8 (CCK-8) assay [21]. At 24 h and 48 h, no cytotoxicity to either cancer (4T1) cells or normal (L02) cells was observed with the Bi@LyP-1 NPs at concentrations below 200 μg mL^−1^. Using the same kit, the cytotoxicity induced by Bi@SR-PEG NPs in 4T1 cells was evaluated. At a concentration of 100 mg L^−1^ and after 24 h of incubation, the mean cell viability was observed to be at a high level (>90%) [29].

The cytotoxicity against the human HT-29 colon cancer cells by the biogenic Bi NPs produced by *Delftia* sp. SFG was evaluated [53]. The IC_50_ value was 28.7 ± 1.4 μg mL. These biogenic Bi NPs induced late apoptosis or necrosis via the activation of oxidative stress independent of the caspase-3 pathway.

The cytotoxic effect was also determined by MTT assay against both lung and breast cancer cell lines and 3T3 cells. The cytotoxicity of the biogenic Bi NPs was measured with an IC_50_ value of 10.9 ± 0.9 μg mL for lung cancer cells and 35.4 ± 0.5 μg mLµM^1^ for breast cancer cells [54]. These Bi NPs showed a higher toxicity on these cells compared to mouse 3T3 fibroblast cells, with an IC_50_ value of 42.8 ± 1.7 μg mLµM^1^.

A cell viability based on MTT assay was performed with BSA-stabilized Bi NPs on the murine macrophage cell line RAW 264.7 (immune system mammal cells) [55]. Cells were exposed to a range of concentration between 0.01 and 50 μg mL^−1^ of Bi NPs during 24 h and 48 h. No effect was observed for 24 h, but a decrease in cell viability was detected after 48 h exposure to Bi NPs concentration greater than 20 μg mL^−1^. A decrease in cell attachment was also observed at 50 μg mL^−1^ of Bi NPs from 24 h exposure. An increase in the phagocytic effect by macrophages and DNA repair foci was also noticed at 50 μg mL^−1^ of Bi NPs at both exposure times.

The viabilities of HUVECs and HeLa cells co-cultured with various concentrations of Bi@mSiO_2_@MnO_2_ (20, 40, 80, 160, 320 µg mL^1^) was evaluated using a standard CCK-8 assay [34]. When the concentration of Bi@mSiO_2_@MnO_2_ NCs reached 320 µg mL µM^1^, the cell viabilities were above 90%, suggesting a quite low cytotoxicity for these Bi NPs. Sixteen days after IV injection of Bi@mSiO_2_@MnO_2_ and PTT treatment, haematoxylin and eosin staining was performed on the main organs and no obvious lesions, inflammation or necrosis were observed, indicating a quite good Bi NP biocompatibility.

Bi NPs produced by laser ablation were stabilized to avoid aggregation by two different approaches: using culture medium supplemented with fetal bovine serum (FBS) or by BSA. Bi NPs cytotoxicity was assessed on BALB/c 3T3 cells using neutral red uptake assay [56]. An IC_50_ value was measured at 28.51 ± 9.96 μg mL^−1^ for Bi NPs stabilized by FBS and 25.54 ± 8.37 μg mL^−1^ for Bi NPs stabilized by BSA, indicating a non-significant difference regardless of the corona composition. A further study to identify the interaction between Bi NPs and cells suggested that, when the Bi NPs were internalized by endocytosis in 3T3 cells (at IC_50_ dose), they altered the cell ultrastructure and cell death caused by apoptosis.

Several human cell lines from different organs, such as the human embryonic kidney 293 cells (HEK293), human hepatocarcinoma (HepG2), human umbilical vein endothelial cells (HUVEC) and human lung adenocarcinoma A549 cells, were tested for cytotoxicity of BSA-stabilised Bi NPs [17]. No viability decrease in HepG2 and HUVEC cells was observed, indicating low toxicity on human liver and blood cells for a Bi NP concentration between 2.5 and 160 μg mL^−1^. However, A549 cell viability decreased to 78% and 55% for HEK293 at the highest concentration of 160 μg mL^−1^, demonstrating a slight and significant cytotoxicity for these two cell types, respectively. Fluorescence staining of monodansylcadaverine (MDC) demonstrated that Bi NPs induced autophagy. Indeed, the increase in MDC-labeled vacuoles correlated with an increased concentration of Bi NPs in the cytoplasm.

The cellular uptake in HEK293 was then quantified by kinetics analysis correlated to fluorescence detection. For that purpose, Bi NPs were labelled with fluorescein isothiocyanate (FITC), introduced by coupling with the amino group on BSA surface (BiNP-FITC). For 24 h, the mean fluorescence intensity was measured and correlated with the increase in Bi NP concentration in cells, confirmed by ICP–MS analysis. This accumulation in intracellular vacuoles was confirmed using TEM and EDX analysis. Finally, the mechanism of uptake in HEK293 cells was investigated using intracellular vacuole markers and endocytic inhibitors, which indicated that Bi NPs required protein trafficking from the endoplasmic reticulum to the Golgi apparatus. This study illustrated that cytotoxicity in HEK293 cells was partially induced by Bi NPs, but also by the autophagy mechanism and cellular uptake of Bi NPs by endocytic mechanism.

BSA-based Bi NPs were evaluated in vitro and in vivo to determine their toxicity in kidney cells (HEK 293) and their protective role in nephrotoxicity elicited by autophagy [57]. After IV injection in mice, blood biochemical analysis showed significant increases in CREA, BUN and total protein in a concentration-dependent manner with Bi NPs, while only a slight increase in potassium was obtained with higher Bi NP concentrations (5 mg kg^−1^). These results showed that Bi NPs can cause acute kidney injury in association with increased levels of autophagy-related proteins LC3II, while the autophagic flux indicator p62 remained unchanged. Then, experiments combined with rapamycin and chloroquine demonstrated that Bi NPs (2.5 mg kg^−1^) induced an attenuated nephrotoxicity and an increased autophagic flux blockage, due to these two treatments, respectively. In vitro experiments using HEK296 cells, treated with Bi NPs (80 μg mL^−1^) for 24 h and analysed by TEM, showed double-membrane vacuoles formation, characteristic of the occurrence of autophagy. Finally, the production of ROS, classical autophagy inducers, was shown to be induced by Bi NPs. To gain further insight, HEK293 cells were also treated with Bi NPs with/without N-acetylcysteine (NAC), a scavenger of ROS, for 24 h. The co-treatment with Bi NPs and NAC reduced Bi NP-induced autophagy, which is initiated by ROS.

The cytotoxicity of Bi@GEL NPs (BGPs) was evaluated in vitro with a MTT assay on HepG-2 and HeLa cells [33]. After 24 h, no obvious cytotoxicity was observed at different concentrations up to 500 μg mL^−1^. In vivo, 15 days after IV injection of BGPs (100 μL, 4 mg mL^−1^), major organs and hematological analyses confirmed low Bi retention. This can be explained by the gradual degradation of BGPs under a pH of 7.2 (around the pH of tumour microenvironment), avoiding long-term toxicity. Thanks to their low cytotoxicity and high clearance from the body, BGPs demonstrated a favorable biosafety profile.

An evaluation of Bi@BSS cytotoxicity was performed on human gingival fibroblast (HGF-1) cells involved in oral mucosa [38]. The tests, using lactate dehydrogenase (LDH) and MTS assays, indicated a low cytotoxicity, with 6% cell membrane damage and with 4% mitochondrial function affected for both assays, respectively. These tests were performed at a highest concentration of 60 μg mL^−1^ after 24 h treatment with Bi@BSS NPs. The Bi NPs were highly internalised, given their small average sizes of 4–22 nm, by endocytosis in HGF-1 cells and agglomerated in the cytoplasm, near the nucleus and the endoplasmic reticulum. According to these results, the Bi@BSS NPs could therefore be potentially used in dental materials and antiseptic solutions because no cytotoxicity was induced at the concentration where these antibacterial agents are effective. However, further investigations are needed to know their toxic effects in vivo.

The biocompatibility and, especially, toxicity evaluation of Bi NPs remains incomplete to consider clinical studies. Most of the studies describe in vitro results; only a few publications describe in vivo partial toxicity assessment. Moreover, comprehensive studies to understand the relationship between the surface coating of Bi NPs and their biodistribution and pharmacokinetics (also depending on the route of administration and the dose) are required.

## 7. Conclusions

The exhaustive analysis of forty-one studies published since 2012 concerning the medical applications of Bi NPs undeniably shows the theranostic interest of these metallic NPs (Table 1). In the diagnostic field, preclinical proofs of concept have been demonstrated for X-ray, photoacoustic and fluorescence imaging. In the therapeutic field, several preclinical studies show the Bi NP’s potential as X-ray radiosensitizers for RT and photothermal agents for NIR-PTT, but also as bactericidal, fungicidal, antiparasitic and antibiofilm agents.

In contrast, the biocompatibility evaluation and, especially, the evaluation of Bi NP’s toxicity remains incomplete. Most studies only describe in vitro results, and few publications mention in vivo toxicity assessment. This literature is far from having established a sufficient enough Bi NP safety profile to consider upcoming clinical studies. It is probably within the realm of a rigorous and complete evaluation of Bi NP’s nanotoxicity that we will now have to develop research work to eventually consider a clinical development of these nano-objects.

## Figures and Tables

**Table 1:** Metallic bismuth nanoparticles as imaging contrast agents and as X-ray radiosensitizers, for theranostic applications.

Entry	Capping Agent	Diameter TEM (nm)	Biological Applications	Proof of Concept	Reference
1	PVP, APTES and conjugation with folic acid	30	X-ray radiosensitizers to detect and kill circulating tumor cells	In vitro	Hossain et al. 2012
2	PVP and conjugation with *Pseudomonas aeruginosa* polyclonal antibody	30	X-ray radiosensitizers to eliminate bacteria	In vitro	Luo et al. 2013
3	Red blood cell membrane and conjugation with folic acid	56	X-ray radiosensitizers for breast cancer	In vitro & in vivo (mice)	Deng et al. 2018
4	Cellulose nanofiber	2–10	X-ray radiosensitizers for breast cancer	In vitro & in vivo (mice)	Jiao et al. 2018
5	1-Dodecanethiol PEGylated phospholipid	40	CT tomography & photothermal and radiotherapy treatment of tumors.	In vitro & in vivo (mice)	Yu et al. 2018
6	DSPE-PEG5000 and conjugation to peptide LyP-1	3.6	CT tomography & photoacoustic imaging agent & NIR-photothermal and radiotherapy treatment of tumors.	In vitro & in vivo (mice)	Yu et al. 2017
7	DLPC (,2-dilauroyl-sn-glycero-3-phosphocholine)	47	CT tomography & photoacoustic imaging agent & NIR-photothermal treatment of tumors.	In vitro & in vivo (mice)	Yang et al. 2018
8	Poly (vinylpyrrolidone)	2.7	CT &photothermal-imaging-guided photothermal therapy	In vitro & in vivo (mice)	Lei et al. 2017
9	Ppy PEG	70	CT tomography & photoacoustic imaging agent & NIR-photothermal treatment of tumors	In vitro & in vivo (mice)	Yang Sisi et al. 2017
10	DSPE PEG	100 ****	CT tomography & photoacoustic imaging agent & NIR-photothermal treatment of tumors	In vitro & in vivo (mice)	Lu et al. 2019
11	GEL, BSA, HSA	15–19	CT tomography & infrared thermal & antitumor PTT	In vitro & in vivo (mice)	Liu et al. 2020
12	PEG	41	Trimodal imaging (CT, photoacoustic and infrared thermal) & antitumor PTT	In vitro & in vivo (mice)	Li et al. 2017
13	d-glucose & 1,2-propanediol	74	CT tomography	In vitro	Brown et al. 2014
14	PLGA and SiO_2_	12	CT tomography	In vitro	Chakravarty et al. 2016
15	PLGA	120 *	CT tomography	In vitro & ex vivo (chicken wing forearm)	Swy et al. 2014
16	PEG NH_2_	4 to 100	CT tomography and fluorescence imaging	In vitro & in vivo (mice)	Bi et al. 2018
17	Polymerized d-glucose	22	CT tomography (GI tract)	In vitro & in vivo (mice)	Wei et al. 2016
18	BSA	6–7	CT tomography, fluorescence imaging and cytotoxicity	In vitro & in vivo (mice)	Liu et al. 2017
19	Surfactant (not described)	10–100 *	CT tomography and radiotherapy	In vivo (mice)	Torisi et al. 2018
20	Mesoporous silica	115 nm ****	CT tomography/magnetic resonance imaging chemo/photothermal/chemodynamic therapy	In vitro & in vivo (mice)	Zhao et al. 2021

* SEM measurement, **** DLS measurement, PLGA = Poly (DL-lactic-co-glycolic acid), PVP = Polyvinylpyrrolidone, APTES = (3-Aminopropyl) triethoxysilane, DSPE-PEG5000 = 1,2-distearoyl-sn-glycero-3-phosphoethanolamine-N-[amino(polyethylene glycol)-5000], PEG NH_2_: N-[amino(polyethylene glycol)], BSA = bovine serum albumin.

**Table 2:** Metallic bismuth nanoparticles as bactericidal, fungicidal, antiparasitic and antibiofilm agents.

Entry	Capping Agent	Diameter TEM (nm)	Biological Applications	Proof of Concept	Reference
1	Citrate	3.3	Antimicrobial activity against *Streptococcus mutans* and inhibition of biofilm formation by *Streptococcus mutans*	In vitro	Hernandez et al. 2012
2	Carboxylic groups	100	Antibacterial activity against *Helicobacter pylori*	In vitro	Nazari et al. 2013
3	Subsalycilate	22, 31, 45 and 58 **	Antibacterial activity against *E. coli*, *P. aeruginosa*, *S. aureus* and *S. epidermidis*	In vitro	Mariela Flores-Castaneda et al. 2015
4	Subsalycilate	4-22	Antibacterial activity against *Actinomyces israelii*, *Aggregatibacter actinomycetemcomitans* serotype b, *Capnocytophaga* *gingivalis*, *Eikenella corrodens*, *Fusobacterium nucleatum* subsp. *nucleatum*, *Parvimonas* *micra*, *Porphyromonas gingivalis*, *Prevotella intermedia*, *Streptococcus mutans* and *Streptococcus sanguinis*	In vitro	Vega-Jimenez et al. 2017
5	PVP	8	Antimicrobial activity and antibiofilm activity on *Staphylococcus aureus* and *Candida albicans*	In vitro	Vazquez-Munoz et al. 2020
6	2,3-dimercapto-1-propanol	28	Antibacterial activity against *Streptococcus mutans* and *Streptococcus gordonii* Antimycotical activity against *Candida albicans* Inhibition of biofilm formation by a multispecies population of *S. mutans*, *L. casei*, *S. gordonii* and *C. albicans*	In vitro	Badireddy et al. 2014
7	2,3-dimercapto-1-propanol	3–15	Antibacterial activity Inhibition of biofilm formation by *Pseudomonas aeruginosa*	In vitro	Badireddy et al. 2014
8	2,3-dimercapto-1-propanol	25 *	Antiparasitic activity Inhibition of *Trichomonas vaginalis* growth	In vitro	Rodríguez-Luis et al. 2017
9	2,3-dimercapto-1-propanol	40	Antibacterial activity of *Enterococcus faecalis* and *Streptococcus salivarius*	In vitro	Azad et al. 2020 Rostamifar et al. 2021
10	Mineral trioxide aggregate supplemented with Bi NPs coated with 2,3-dimercapto-1-propanol	ND	Antibacterial, antifongical activity of *Enterococcus faecalis*, *Escherichia coli*, and *Candida albicans* Inhibition of biofilm formation by *Enterococcus faecalis* and mechanical properties of mineral trioxide aggregate (MTA) supplemented with Bi NPs	In vitro	Hernandez-Delgadillo et al. 2017
11	ND	40	Antimicrobial activity of *Campylobacter jejuni* Pl—09.c, *Listeria monocytogenes* ATCC 19112, *Yersinia enterocolitica* 12/15- 08, *Salmonella typhimurium* N°16, *Escherichia coli* N°4; *Mycoplasma* *arginini* G 230, *Acholeplasma laidlawii* ATCC 23206, *Bacillus anthracis* M-71, *Leptospira Pomona* microorganisms	In vitro	Rieznichenko et al. 2015

PVP = Polyvinylpyrrolidone, ND: not determined, ** SAXS measurement, * SEM measurement.

**Table 3:** Biocompatibility and toxicity of metallic bismuth nanoparticles.

Entry	Capping Agent	Diameter TEM (nm)	Proof of Concept	Cytotoxicity	Toxicity In Vivo	Reference
1	Cellulose nanofiber	2–10	In vitro & in vivo (mice)	MTT assay: 4T1 breast cancer cells	ND	Jiao et al. 2018
2	1-Dodecanethiol PEGylated phospholipid	40	In vitro & in vivo (mice)	kit-8 (CCK-8) assay: 4T1 cancer cells	Toxicity on mice (IV injection): hematology & junctional liver and kidney markers	Yu et al. 2018
3	DSPE-PEG5000 and conjugation to peptide LyP-1	3.6	In vitro & in vivo (mice)	kit-8 (CCK-8) assay: 4T1 cancer cells and hepatic L02 cells	ND	Yu et al. 2017
4	DLPC (,2-dilauroyl-sn-glycero-3-phosphocholine)	47	In vitro & in vivo (mice)	MTT assay: MDA-MB-231 and MCF-10A cells	Body weight changes, histology of main organ and blood analysis	Yang et al. 2018
5	Poly (vinylpyrrolidone)	2,7	In vitro & in vivo (mice)	MTT assay: U14 cells	Histology of main organ and blood analysis	Lei et al. 2017
6	Ppy PEG	70	In vitro & in vivo (mice)	kit-8 (CCK-8) assay: HUVEC & 4T1 cancer cells	Histology of main organ and serum biochemistry	Yang Sisi et al. 2017
7	DSPE PEG	100 ****	In vitro & in vivo (mice)	MTT assay: C6 & Cos-7 cells	Histology of main organ, liver function and blood analysis	Lu et al. 2019
8	GEL, BSA, HSA	15–19	In vitro & in vivo (mice)	MTT assay: HepG-2 & HeLa cells	Toxicity on nude mice (IV injection): hematology & major organs	Liu et al. 2020
9	PEG	41	In vitro & in vivo (mice)	kit-8 (CCK-8) assay: HUVEC & HeLa cancer cells hemolytic behavior on Red Blood Cell	Histology of main organ and serum biochemistry	Li et al. 2017
10	d-glucose & 1,2-propanediol	74	In vitro	MTS assays: HeLa cell & J774.A macrophage cell	ND	Brown et al. 2014
11	PLGA and SiO_2_	12	In vitro	MTT assay: Raw264.7 macrophage	ND	Chakravarty et al. 2016
12	PLGA	120 *	In vitro & ex vivo (chicken wing forearm)	MTT assay STO mouse fibroblast	Toxicity on rats (IV & IP injection): hematology & Blood chemistry, abnormalities & pathological & histopathological analysis	Swy et al. 2014
13	PEG NH_2_	4 to 100	In vitro & in vivo (mice)	MTT assay: L929 cells	ND	Bi et al. 2018
14	Polymerized d-glucose	22	In vitro & in vivo (mice)	MTT assay: HeLa cells	Toxicity on mice (oral administration) blood chemistry & histopathological analysis	Wei et al. 2016
15	Subsalycilate	4-22	In vitro	LDH and MTS assays in Human gingival fibroblast (HGF-1) cells	ND	Vega-Jimenez et al. 2017
16	Mineral trioxide aggregate supplemented with Bi NPs coated with 2,3-dimercapto-1-propanol	ND	In vitro	Human gingival fibroblasts (HGF)	ND	Hernandez-Delgadillo et al. 2017
17	PEG-SiO_2_, NH_2_-SiO_2_, SiO_2_ or PVP	20	In vitro	MTT, G6PD and calcein AM/EthD-1 assays: HeLa, MG-63	ND	Luo et al. 2012
18	PVP	78 ***	In vitro	Breast cancer cell line MCF-7	ND	Hammood et al. 2016
19	2,3-dimercapto-1-propanol	18	In vitro	Erythrocytes, leukocytes, & neutrophils	ND	Hernandez-Delgadillo et al. 2015
20	2,3-dimercapto-1-propanol	18	In vitro	Monkey kidney cells	ND	Hernandez-Delgadillo et al. 2016
21	2,3-dimercapto-1-propanol	24 *	In vitro & in vivo (mice)	HeLa, DU145, HCT-116	Histology of main organ	Cabral-Romero et al. 2020
22	2,3-dimercapto-1-propanol	28 *	In vitro	Human breast cancer cell line MCF-7	ND	Hernandez-Delgadillo et al. 2018
23	ND	40–120	In vitro	MTT assay: HT29 cells	ND	Shakibaie et al. 2018
24	ND	20-120	In vitro	MTT assay: lung (A549), breast (MCF-7) cancer cells, 3T3 fibroblast cells	ND	Shakibaie et al. 2018
25	BSA	4–87 ****	In vitro	Murine macrophage cell line RAW 264.7	ND	Zablocki da Luz et al. 2020
26	BSA	20	In vitro	BALB/c 3T3 cells	ND	Reus et al. 2018
27	BSA	6-7	In vitro & in vivo (mice)	MTT assay: HEK293, HepG2, HUVEC, A549 cells	Mouse mammary carcinoma cell line 4T1 (IV injection)	Liu et al. 2017
28	BSA	6–7	In vitro & in vivo (mice)	HEK223 cells	Histology of kidney and nephrotoxicity on Balb/c mice (IV injection)	Liu et al. 2018
29	Mesoporous silica	115 nm ****	In vitro & in vivo (mice)	CCK-8 assay HUVECs and HeLa cells	Body weight changes, histology of main organ	Zhao et al. 2021

ND: not determined, PVP = Polyvinylpyrrolidone, * SEM measurement, *** AFM measurement, **** DLS measurement, PLGA = Poly (DL-lactic-co-glycolic acid), DSPE-PEG5000 = 1,2-distearoyl-sn-glycero-3-phosphoethanolamine-N-[amino(polyethylene glycol)-5000], PEG NH_2_: N-[amino(polyethylene glycol)], BSA = bovine serum albumin.

## Data Availability

Not applicable.

## Abbreviations

ALT	Alanine aminotransferase
ALP	Alkaline Phosphatase
AST	Aspartate aminotransferase
Au NPs	Gold nanoparticles
Bi NPs	Metallic bismuth nanoparticles
BSA	Bovine serum albumin
BSS	Bismuth subsalicylate
BUN	Blood urea nitrogen
CCK-8	Cell counting kit-8
CPTT	Chemo-photothermal therapy
CREA	Creatinine
CT	Computed tomography
DLPC	1,2-dilauroyl-sn-glycero-3 phosphocholine
DSPE	2-distearoyl-sn-glycero-3-phosphoethanolamine
FBS	Fetal Bovine Serum
FITC	Fluorescein isothiocyanate
G	GANEX
GI	Gastrointestinal
HGFs	Human gingival fibroblasts
HU	Hounsfield units
IRT	Infrared thermal
LDH	Lactate dehydrogenase
MDC	Monodansylcadaverine
MIC	Minimal inhibitory concentration
MTA	Mineral Trioxide Aggregate
NAC	*N*-acetylcysteine
NIR	Near infrared
PA	Photoacoustic
PAI	Photoacoustic imaging
PEG	Polyethylene glycol
PLGA	Poly (DL-lactic-co-glycolic acid)
PPy	Polypyrrole
PTT	Photothermal therapy
PVP	Poly (vinylpyrrolidone
RBC	Red blood cell
ROS	Reactive oxygen species
RT	Radiotherapy
SPR	Surface plasmon resonance
UA	Uric acid
XCAs	X-ray contrast agents
